# Atypical Self-Focus Effect on Interoceptive Accuracy in Anorexia Nervosa

**DOI:** 10.3389/fnhum.2016.00484

**Published:** 2016-09-27

**Authors:** Olga Pollatos, Beate M. Herbert, Götz Berberich, Michael Zaudig, Till Krauseneck, Manos Tsakiris

**Affiliations:** ^1^Clinical and Health Psychology, Institute of Psychology and Education, Ulm UniversityUlm, Germany; ^2^Department of Psychology, Clinical Psychology and Psychotherapy, Eberhard Karls University of TübingenTübingen, Germany; ^3^Klinik WindachWindach, Germany; ^4^kbo-Isar-Amper-Klinikum gemeinnützige GmbHMunich-Haar, Germany; ^5^Laboratory of Action and Body, Department of Psychology, Royal Holloway, University of LondonEgham, UK

**Keywords:** anorexia nervosa, interoceptive accuracy, self-focused attention, body dissatisfaction, heartbeat perception, cognitive-behavioral therapy

## Abstract

**Background**: Interoceptive abilities are known to be affected in anorexia nervosa (AN). Previous studies could show that private self-focus can enhance interoceptive accuracy (IAcc) in healthy participants. As body dissatisfaction is high in AN, confrontation with bodily features such as the own face might have a directly opposed effect in AN. Whether patients with AN can benefit from self-focus in their IAcc and whether this pattern changes over the time-course of cognitive behavioral therapy was investigated in this study.

**Methods**: Fifteen patients with AN from the Psychosomatic Clinic in Windach were assessed three times in the time course of a standardized cognitive-behavioral therapy. They were compared to 15 controls, recruited from Ulm University and tested in a comparable setting. Both groups performed the heartbeat perception task assessing IAcc under two conditions either enhancing (“Self”) or decreasing (“Other”) self-focused attention. Furthermore, body dissatisfaction was assessed by a subscale of the Eating Disorder (ED) Inventory 2.

**Results**: Patients with AN scored higher in IAcc when watching others’ faces as compared to one’s own face while performing the heartbeat perception task. The opposite pattern was observed in controls. IAcc remained reduced in AN as compared to controls in the time-course of cognitive-behavioral therapy, while body-dissatisfaction improved in AN. High body dissatisfaction was related to poorer IAcc in the “Self” condition.

**Conclusions**: Our findings suggest that using self-focused attention reduces IAcc in AN while the opposite pattern was observed in controls. Confronting anorexic patients with bodily features might increase body-related avoidance and therefore decrease IAcc. The current study introduces a new perspective concerning the role of interoceptive processes in AN and generates further questions regarding the therapeutic utility of methods targeting self-focus in the treatment of AN.

## Introduction

Anorexia nervosa (AN) is a psychiatric disorder defined by excessive weight loss, fear of gaining weight, a disturbed body image and a rejection of the own body (Klein and Walsh, 2003). Friederich et al. (2010) describe body dissatisfaction as an important precipitating and maintenance factor in AN. Interestingly, the perceptual disturbance of one’s own body is highlighted as important for the pathophysiology of AN (Friederich et al., 2010). Friederich et al. (2010) suggest that observed hyperactivation in insula along with hypoactivation in anterior cingulate may be critical for altered interoceptive processes involved in body self-comparisons in AN as both insula and anterior cingulate are central structures associated with the processing of interoceptive signals (see e.g., Craig, 2003; Critchley et al., 2004; Seth et al., 2012). This is in accordance to previous studies (Fassino et al., 2004; Matsumoto et al., 2006; Pollatos et al., 2008; Klabunde et al., 2013) showing that patients with eating disorders (ED) and especially patients with AN have difficulties in interoceptive functions assessed with different methods, e.g., when using questionnaires or behavioral tests targeting accuracy in detecting internal signals such as heartbeat detection.

Interoception is the body-to-brain axis of sensation concerning the state of the internal body and its visceral organs (Cameron, 2001; Craig, 2002). The generation and perception (interoception) of internal states of bodily arousal are central to many theoretical accounts of emotion (e.g., James, 1884; Damasio, 1999). As a general concept, interoception includes two forms of perception: proprioception (signals from the skin and musculoskeletal apparatus) and visceroception (signals from the inner organs like heart rate, breath and hunger). Garfinkel and Critchley (2013) first emphasized the importance to differentiate between different facets of interoceptive processing, suggesting to distinguish between interoceptive accuracy (IAcc; e.g., behavioral testing such as performance on heartbeat perception tests), metacognitive awareness (e.g., confidence-accuracy correspondence) and subjective interoceptive sensibility (e.g., as assessed via self-report questionnaires, e.g., body perception questionnaire). In former research these different levels were often used in an interchangeable way which could have contributed to diverging results.

Referring to a recent study of Garfinkel et al. (2015) IAcc might be the core ability within the construct of interoception underpinning other interoceptive measures. Individuals differ substantially in measures of IAcc, the ability to perceive consciously signals arising from the body. Measuring a person’s ability to perceive and accurately report one’s heartbeats at rest is often used to quantify these differences (Schandry, 1981; Cameron, 2001; Critchley et al., 2004; Pollatos and Schandry, 2004; Pollatos et al., 2005; Dunn et al., 2007). First evidence indicates that a focus on self-related stimuli can manipulate IAcc suggesting a dynamic relationship between self-awareness and interoception. IAcc can be improved when persons attend to their self as operationalized with looking in the mirror (Ainley et al., 2012). This effect was most pronounced in persons with low IAcc at the baseline condition. Similar results were achieved when persons paid attention to bodily and narrative aspects of the self (Ainley et al., 2013).

Another set of studies has looked at the role of IAcc in body-awareness using various established paradigms of bodily illusions that have been shown to alter the sense of body-ownership. For example, it has been demonstrated that interoceptive processes modulate the integration of multisensory body percepts as shown by Tsakiris et al. (2011) and Suzuki et al. (2013). Further research has also demonstrated that interoceptive signals can also be used for inducing bodily illusions as cardio-visual stimulation was associated with an affected sense of self in one patient before and after insula resection surgery (Ronchi et al., 2015). Interoceptive influences extend from the basic levels of multisensory integration to the conscious attitudes that we hold about our body, highlighting the role that interoception potentially plays across different hierarchical levels of body-representations. Pollatos et al. (2008) have shown that patients with ED show reduced IAcc relative to controls. Ainley and Tsakiris (2013) recently showed an inverse relation between levels of IAcc and self-objectification (Ainley and Tsakiris, 2013), suggesting that better IAcc is associated with a lower tendency to experience one’s body as an object. The body as object describes an attitude of evaluation of its appearance and a position as if seen through the eyes of others. Similarly, a negative relation has been shown between IAcc and body-image dissatisfaction (Emanuelsen et al., 2015) in a sample of 82 high school students (mean age 17). Of relevance, other studies have reported that levels of IAcc influence eating habits, e.g., IAcc is inversely related to intuitive eating (Herbert et al., 2013). Although these findings are suggestive of the role that interoception may have for body-image satisfaction and related behaviors (e.g., eating), the question of how experimentally manipulating self-focus may change IAcc in AN, as it does in healthy individuals has not been examined before.

A negative evaluation of one’s own body is often associated with body-related avoidance (e.g., not looking in the mirror or hiding one’s body under baggy clothes, see Trautmann et al., 2007). Therefore, it is an open question whether self-focus using bodily features can indeed improve IAcc in AN as demonstrated in healthy participants. The role of body-dissatisfaction in this context has not been elucidated so far. The aim of this study was to investigate possible changes in IAcc using a paradigm manipulating the self-focus during the interoceptive task. Anorexic patients from the psychosomatic clinic Windach am Ammersee were examined three times during stationary therapy (first week of clinic stay, 4–6 weeks later respectively after a gain of 2 body mass index (BMI) points, and before their dismissals at the end of their therapy) and compared to healthy controls. We were primarily interested in testing whether AN patients will benefit in IAcc from self-focus in the same extent as healthy controls, as well as if IAcc is improved over time by cognitive-behavioral therapy.

## Materials and Methods

### Participants

Female patients with current AN were recruited from the Psychosomatic Clinic Windach am Ammersee. Reflecting clinical routine, diagnoses were determined according to International Classification of Disease 10 criteria based on semi-structured clinical interviews administered by a senior staff member. The patients took part in a cognitive behavioral therapy with special attention to maladaptive emotional processes and the systemic context. They agreed with the therapists on a target weight and a weight gain of 700 g per week.

Data for this study were collected in a longitudinal design targeting IAcc under two conditions: looking at the own face (condition “Self”) and looking at another face (of an unknown person; condition “Other”) while the heartbeat perception task was carried out (details see below). Body weight and height were assessed at the end of each session. Participants were tested three times based on the therapy-process at the beginning (T1), after 4–6 weeks respectively after an increase of 2 BMI points (T2) and at the end of therapy (T3). On average, patients stayed in the clinic 12–14 weeks and were included in the study in the first or second week of their therapy. Fifteen women with AN were included in the experiments. Mean age in the AN group was 27.4 years (SD = 7.8) and mean BMI was 15.7 (SD = 1.3) at T1. Exclusion criteria were any purging at the moment or former diagnosis of bulimia nervosa.

Fifteen female healthy controls were recruited from staff or students at the Ulm University and matched according age and educational background. They received a compensation of €20. Controls had a mean age of 27.9 (SD = 7.6) and a mean BMI of 21.0 (SD = 1.8). None of them were taking medication (except of contraceptives), had a past or current ED or any other psychiatric or severe somatic illness as assessed by anamnestic questionnaire. Both groups did not differ significantly concerning age (*t*_(df = 28)_ = 0.19, *p* = n.s.) and educational level (educational level assessed by a scoring system for the German school system: (1) without educational qualification; (2) secondary general school certificate; (3) intermediate school certificate; (4) entrance qualification for technical college; (5) entrance qualification for university; AN: mean 3.13 (SD = 1.0); controls: mean 3.4 (SD = 0.9); *t*_(df = 28)_ = −0.74, *p* = n.s.). The study was conducted in accordance with the Declaration of Helsinki, ethical approval was obtained from an institutional review board. Prior to testing, informed consent was obtained.

### Instruments

A short questionnaire explored health status and personal data (e.g., age, educational background). Different standard psychological questionnaires were applied including the subscale “body dissatisfaction” from the ED-Inventory-2 (Garner, 1984). Questions are rated on a 6-point scale, ranging from 1 (never) to 6 (always). High scores indicate higher body dissatisfaction.

IAcc was assessed by a heartbeat perception task in two counterbalanced conditions: looking at the own (“self”) or looking at another face (“other”; a non-familiar female face) while doing the heartbeat perception task. Condition “self” was realized by using a laptop camera focusing on the face of the participant, while during the “Other” condition participants watched a pre-recorded video of a female model (age of the model 21, 24, 26 years; BMI within normal range: 22.6 kg/m^2^, and 20.8 kg/m^2^, 20.5 kg/m^2^) who was looking directly into camera. There were three different female models used, so that for each time point (T1, T2, T3) another pre-recorded video was presented. The order of the models was randomized. Participants were instructed to attentively watch either “Self” or “Other” during the following heartbeat perception tasks. For each condition three heartbeat counting trials of the Mental Tracking Method were used as proposed by Schandry (1981). The three trials per conditions were presented in a random order across participants. A short training interval of 15 s was followed by four intervals of 25, 45 and 35. Participants were asked to count their own heartbeats silently and to verbally report the number of counted heartbeats at the end of each counting phase. The beginning and the end of the counting intervals were indicated by the supervisor. During heartbeat counting, participants were instructed not to take their own pulse or attempt to use other forms of manipulation in order to support counting of their heartbeats. Furthermore, they did not receive any information about the length of the counting phases or the quality of their performances.

IAcc was calculated as the mean heartbeat perception score according to the following transformation:

$$\frac{1}{3}\sum (1-\frac{\left(\left|\text{recorded heartbeats}-\text{ counted heartbeats}\right|\right)}{\text{recorded heartbeats}})$$

IAcc scores range from 0 to 1. Higher scores indicate small differences between the counted and recorded heartbeat and consequently a better IAcc. Other experimental paradigms (e.g., emotional picture presentation and evaluation, attention task) conducted later are not reported here. Each session lasted about 45 min.

### Procedure

Patients were informed about the study by staff and they received written information about the experiment. At each point of data collection, patients were tested individually in a separate, quiet room of the clinic. Controls were examined at the laboratories of the Clinical and Health Psychology department in Ulm. Patients were tested three times based on the therapy-process at the beginning (T1), after 4–6 weeks (T2) and at the end of therapy (T3). Controls were also tested three times using a comparable timetable and setting.

Patients and controls filled in the questionnaires prior to each testing session. Then the assessment of IAcc took place under two conditions. Therefore, cardiac activity was recorded using the mobile heart frequency monitor RS800CX (Polar Electro Oy, Kempele, Finland). The RS800CX is easy to use, non-invasive and -reactive recording of inter-beat-intervals whose validity and reliability compared to alternative ECG measurement devices are established (Koch and Pollatos, 2014a,b).

### Data Analyses

Data analyses were performed with the program SPSS (version 22). Referring to questionnaire and BMI data, repeated measurements ANOVAs were calculated with the factors Group (AN, controls) and Time (T1, T2, T3). Furthermore, IAcc was examined with the factors Group (AN, controls), Time (T1, T2, T3) and Condition (Self, Other). Pearson correlation analyses were carried out between body dissatisfaction scores and IAcc during the “self” and “other” condition at T1, T2 and T3. With respect to the correlation analyses, we used Bonferroni correction to adjust the alpha errors for multiple comparisons. Statistical significance levels reported correspond to *p*-values less than 0.05, 0.01 and 0.001, respectively. In the “Results” Section, uncorrected *F*-values are reported together with the Greenhouse-Geiser epsilon values and corrected degrees of freedom.

## Results

### Sample Description and Questionnaire Data

The relevant sample characteristics obtained from both participant groups concerning BMI and body dissatisfaction are shown in Table 1. Results of the repeated measurements ANOVAs are also summarized there.

**Table 1:** **Body mass index (BMI) and body dissatisfaction during the time course of therapy contrasting anorexic patients (*N* = 15) and controls (*N* = 15)**.

	T1 Mean (SD)		T2 Mean (SD)		T3 Mean (SD)	
	Anorexics	Controls	Anorexics	Controls	Anorexics	Controls
BMI (kg/m^2^)	15.72 (1.27)	21.19 (1.79)	16.93 (1.29)	21.17 (1.85)	18.25 (0.98)	21.11 (1.86)
ANOVA	Time *F*_(2,56)_ = 81.03; *p* < 0.001; *η*^2^ = 0.74; *ε* = 1.00					
	Time × Group: *F*_(2,56)_ = 93.33; *p* < 0.001; *η*^2^ = 0.77; *ε* = 1.00					
	Group: *F*_(1,28)_ = 57.46; *p* < 0.001; *η*^2^ = 0.67; *ε* = 1.00					
Body dissatisfaction (range 1 –6)	4.38 (0.87)	2.96 (0.94)	3.93 (0.89)	2.90 (1.02)	3.51 (1.18)	2.95 (1.01)
ANOVA	Time: *F*_(2,56)_ = 6.67; *p* < 0.01; η^2^ = 0.19; *ε* = 0.85					
	Time × Group: *F*_(2,56)_ = 6.11; *p* < 0.01; *η*^2^ = 0.18; *ε* = 0.83					
	Group: *F*_(1,28)_ = 9.06; *p* < 0.01; *η*^2^ = 0.24; *ε* = 0.83					

BMI significantly increased in AN patients only; BMI of AN patients always was smaller than the BMI of controls (at all time points T1–T3; *p*s < 0.001). Only AN patients exhibited a decrease in body dissatisfaction over time; differences to controls were significant for T1 (*p* < 0.001) and T2 (*p* < 0.01), but not for T3 (*p* = 0.17).

### Interoceptive Accuracy

**Figure 1:**
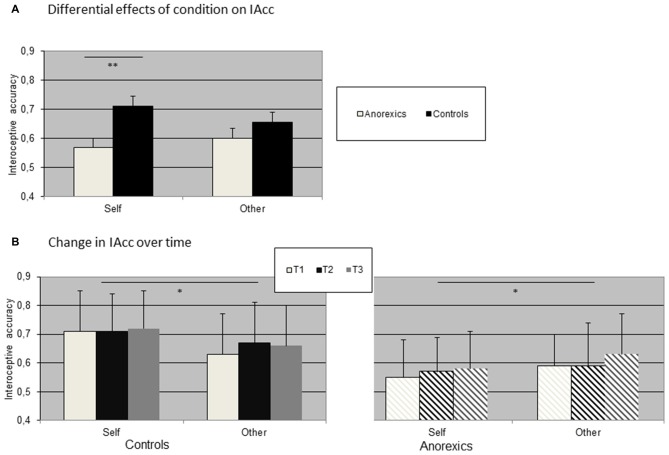
**Distribution of interoceptive accuracy (IAcc) contrasting controls and anorexics in (A) both conditions and (B) over time.** (Bars represent standard error of means, SEM; *p < 0.05; **p < 0.01). **(A)** Differential effects of condition on IAcc. **(B)** Change in IAcc over time.

The mean obtained heartbeat perception scores for the two conditions averaged across all time points (Figure 1A) as well as contrasting both groups at time points T1, T2 and T3 (Figure 1B) are summarized in Figure 1.

We observed a significant interaction effect Condition × Group (*F*_(1,28)_ = 10.92; *p* < 0.01; *η*^2^ = 0.28; *ε* = 0.89) as well as a significant main effect Group (*F*_(1,28)_ = 5.13; *p* < 0.05; *η*^2^ = 0.16; *ε* = 0.59). While mean IAcc was higher for controls (mean 0.68) as compared to anorexic patients (mean 0.58), separate ANOVAs for each group showed that in controls IAcc during the condition “Self” was always higher as compared to “Other”(Condition × Time (*F*_(1,14)_ = 6.18; *p* < 0.05; *η*^2^ = 0.28; *ε* = 0.80). The opposite effect was observed for anorexic patients (Condition × Time (*F*_(1,14)_ = 4.96; *p* < 0.05; *η*^2^ = 0.31; *ε* = 0.64). The main effects Time were not significant in both groups.

### Correlations Between Body Dissatisfaction and IAcc During “Self” Condition

**Figure 2:**
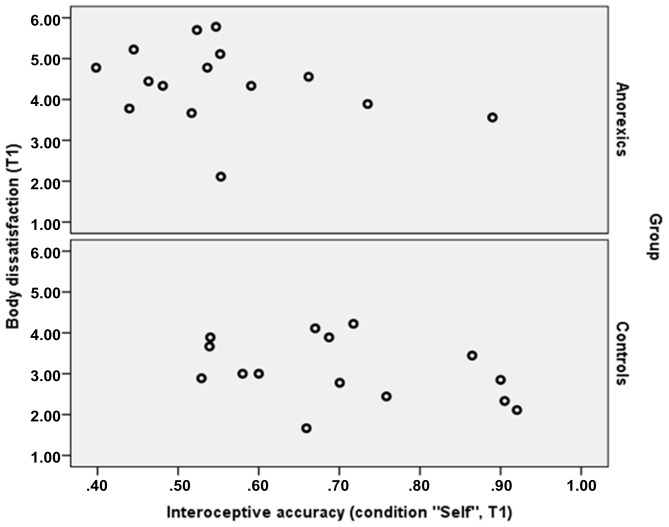
**Distribution of IAcc (condition “Self”) and body dissatisfaction contrasting controls and anorexics at T1**.

In a last step we correlated IAcc during “self” and “other” with mean body dissatisfaction score obtained from questionnaire (*N* = 30, total sample). Due to multiple comparisons, we corrected the alpha error accordingly (*p* values smaller 0.008 are considered significant). We observed significant inverse correlations between IAcc during “Self” condition and body dissatisfaction at T1 (*r* = −0.49, *p* = 0.006) and T2 (*r* = −0.53; *p* = 0.002), while all other correlation coefficients were also inverse, but substantially smaller and did not reach significance (“Self” at T3: *r* = −0.33, *p* = 0.07; “Other” at T1: *r* = −0.37, *p* = 0.04; T2: *r* = −0.33, *p* = 0.07; T3: *r* = −0.35, *p* = 0.06). To compare the distribution between both groups, we plotted the scatter plots between IAcc during the condition “Self” at T1 contrasting anorexics and controls (see Figure 2).

## Discussion

The aim of the present study was to investigate whether AN patients benefit in IAcc from self-focus in the same extent as healthy controls, and whether possible differences change in the time course of an inpatient cognitive-behavioral therapy. In line with former research (Pollatos et al., 2008), anorexic patients exhibited a reduced IAcc averaged across both conditions. Furthermore, IAcc remained reduced in AN during the time course of cognitive-behavioral therapy compared to controls. Remaining deficient IAcc signaling disturbed processing of bodily signals may represent an ongoing risk factor for maintenance of AN. Recent studies using mindfulness-based methods focusing on the body in a non-judging way such as the so-called body-scan could show that interoceptive sensibility as assessed by questionnaire could be improved when trained over a time period of 3 months in healthy controls (Bornemann et al., 2015). Farb et al. (2013) also reported an increase in neural plasticity in interoceptive network structures after daily practices of contemplative techniques such as breath monitoring. Whether these techniques could improve deficits in IAcc using them together with cognitive-behavioral therapy is a relevant future research question. One can assume that observed deficits could be transferred to the perception of bodily signals in general, including accuracy of bodily signals such as hunger and satiety as observed in healthy participants (Herbert et al., 2012). Recent studies also suggest that deficient IAcc might contribute to social problems in healthy populations, demonstrating that higher IAcc is associated with better coping of social exclusion (Werner et al., 2013; Pollatos et al., 2015) and a higher sensitivity to emotions of others (Terasawa et al., 2014). Whether this is also the case in AN needs further evaluation.

Furthermore, AN patients demonstrated differences in the processing of stimuli enhancing self-focus compared to healthy controls: while in accordance to former studies (Ainley et al., 2013) controls showed higher IAcc when watching their face during heartbeat perception, anorexics scored lower when watching their own face as compared to another person’s face. As body dissatisfaction was higher in AN with most pronounced differences at T1, one might assume that the observed atypical lack of a self-observation enhancement effect in IAcc could be related to higher degrees of body-dissatisfaction in the AN group. As we did not ask our participants to evaluate their own face in the experimental situation, we can only speculate that this stimulus is seen as critical as other parts of the body in anorexic females, which then leads to an avoidance of attention on general aspects of the body including interoceptive signal processing. Supporting this interpretation, Trautmann et al. (2007) demonstrated that the own face is a stimulus associated with high avoidance in AN, and also other studies reported alterations in brain activation in anorexics for bodily (see e.g., Uher et al., 2005; Sachdev et al., 2008; Blechert et al., 2010; Miyake et al., 2010). It is an open question whether other methods inducing a self-focus such as self-related words or imagination of positive autobiographic episodes could facilitate IAcc in AN as shown in healthy participants (Ainley et al., 2012, 2013), which could be a promising avenue for future therapeutic methods.

In accordance to Emanuelsen et al. (2015) who showed that body dissatisfaction is related to IAcc in healthy persons, we also observed inverse correlations between IAcc (during “Self”) and body dissatisfaction in this study. As depicted in Figure 2, the pattern of relationship was quite similar both in the groups of anorexics and in the control group at T1, highlighting that the observed results are comparable between controls and patients, though due to the small sample size more data are needed to support this result. It is important to note that the fact that IAcc did not change over the course of therapy, though body dissatisfaction improved, signals that deficient IAcc may represent an independent and stable factor of AN associated with ongoing symptoms and characteristic features of AN, that is not touched by state-of-the-art cognitive behavioral therapies. This also suggests further mechanisms underlying deficient IAcc in AN going beyond body dissatisfaction. Future research could use experimental designs or longitudinal data to examine whether theories of objectification claiming that an evaluative third person view of the body leads to decreased interoceptive abilities (Frederickson and Roberts, 1997; Emanuelsen et al., 2015) or the alternative causal chain suggesting that low levels of IAcc might cause high self-objectification (Ainley and Tsakiris, 2013) are valid.

We suggest that our results highlight a lack of self-focus effect on interoceptive processes in AN, interpreted as dysfunctional integration of bodily information. As known from other studies, lower IAcc is associated with a higher malleability of body-representations (Tsakiris et al., 2011) which was also demonstrated for AN using different experimental paradigms (see e.g., Eshkevari et al., 2012, 2014; Keizer et al., 2014). The atypical pattern of self-focus on IAcc might be interpreted as additional evidence that the dynamic modulation of interoceptive abilities is affected in AN. As we did not assess other aspects of interoception such as confidence in one’s perception, we can only speculate whether the different levels of interoceptive processing respectively the interplay between those levels is affected in AN. Supporting this idea, a recent study by Pollatos and Georgiou (2016) observed such an abnormal overlap between different levels of interoceptive signal processing in bulimic patients. Our observation can be interpreted as potential risk configuration for processes related to a higher malleability of interoceptive signal processing and evaluation of interoceptive signals in AN.

We conclude that anorexic patients, unlike healthy controls, show a significant decrease in their IAcc during self-focus. Limitations of the current study are referred to the small sample of AN patients examined that did also not allow to split into groups for the correlational analyses, and the fact that other facets of interoceptive processes, such as subjective feelings and thoughts of one’s body and interoceptive sensations and metacognitive beliefs, were not systematically addressed. So far, our results questions methods confronting anorexic patients with their body before improving body satisfaction as using bodily stimuli might be associated with greater avoidance and a higher malleability of body-representations in AN as reflected by a decrease in IAcc. Future research highlighting longitudinal data and exploring more facets of interoceptive processes would help understand the pattern observed in AN.

## Author Contributions

OP, MT, TK, MZ and GB substantially contributed to conception, design and acquisition of the data. OP analyzed the data. OP, BMH, MT and GB interpreted the data and drafted the manuscript. All authors approved the version submitted.

## Conflict of Interest Statement

The authors declare that the research was conducted in the absence of any commercial or financial relationships that could be construed as a potential conflict of interest.
